# Extracurricular activity profiles and wellbeing in middle childhood: A population-level study

**DOI:** 10.1371/journal.pone.0218488

**Published:** 2019-07-10

**Authors:** Eva Oberle, Xuejun R. Ji, Carly Magee, Martin Guhn, Kimberly A. Schonert-Reichl, Anne M. Gadermann

**Affiliations:** 1 School of Population and Public Health (The Human Early Learning Partnership), The University of British Columbia, Vancouver, British Columbia, Canada; 2 Department of Educational and Counseling Psychology, and Special Education, The University of British Columbia, Vancouver, British Columbia, Canada; Universidad Nacional de Educacion a Distancia (UNED), SPAIN

## Abstract

This study examined profiles of participation in extracurricular activities (ECAs) in 4^th^ grade children (*N* = 27,121; *Mean age* = 9.20 years; *SD* = .54; 51% male) in British Columbia, Canada. Latent class analyses were used to establish activity profiles and determine class membership; ANCOVA was used to investigate differences in mental wellbeing (optimism, life satisfaction, self-concept) and perceived overall health between groups. Data came from a cross-sectional, population-level child self-report survey (i.e., the Middle Years Development Instrument) implemented with 4^th^ grade children in public schools. We found four distinct ECA profiles: participation in “All Activities”, “No activities”, “Sports” (i.e., individual and team sports), and “Individual activities” (i.e., educational programs, arts/music, individual sports). Wellbeing and health scores were highest for children in the “All Activities” and the “Sports” clusters, and lowest for those in “No Activities” and the cluster reflecting individual activities (i.e., “Individual activities”). Results are discussed in the context of previous research, and with respect to practical relevance.

## Introduction

There has been much interest in understanding the ways in which children and youth spend their time after school, and how these experiences are associated with developmental outcomes [1]. This interest has been spurred, in part, by changing family employment patterns. For instance, in 2014, 69% of Canadian couples with at least one child under the age of 16 were dual earner families (compared to 36% in 1976) and 20% of children lived with a single parent who was employed (compared to 9% in 1979) [2]; trends in the United States are comparable. These demographic changes have raised important questions: Where, how, and with whom are school-aged children spending their after-school time? and to what extent are the activities pursued during these hours linked to developmental outcomes? Extracurricular activities (ECAs), such as sports, the arts, and community programs are of particular interest in this context because they provide adult supervision for children and have been identified as an important setting for fostering children’s and adolescents’ positive development during after-school hours [3–6].

The present study focuses on participation in ECAs after school in a population of 4^th^ grade students in British Columbia (BC), Canada. Our goal was to identify profiles of extracurricular participation, and to investigate whether membership in an extracurricular profile group was associated with differences in children’s wellbeing (i.e., optimism, satisfaction with life, self-concept) and their perceived overall health. Our research builds on previous studies that have identified links between participation in ECAs and a range of positive developmental outcomes. For example, in research with children and adolescents in the United States, extracurricular engagement was positively related to academic performance [7], motivation [8,9], social skills [10], and educational resilience [11], and negatively related to behavioral problems [12], and school dropout [13,14].

The present study extends past research on ECAs in several ways. Most research on ECAs has focused on the developmental period of adolescence and less is known about the benefits of extracurricular engagement for younger children [15]. Middle childhood—typically defined as the age period from 6 to 12 years [16]–is the gateway between childhood and early adolescence and a time during which developmental trajectories are shaped. Understanding participation in ECAs in relation to wellbeing and health during this time period can provide valuable insights into ways to foster positive development before the onset of early adolescence. Further, much research has investigated the competency-enhancing qualities of single types of after-school activities (e.g., sports), whereas fewer studies have taken a person-centered approach to identify profiles that describe combinations of different activities in which young people engage [17,18]. When using a person-centered approach, participation in different types of activities is captured, individuals who pursue the same combinations of activities are sorted into clusters, and cluster-membership can then be linked to developmental outcomes [19]. Finally, this study was conducted at a population level. Population-level research on ECAs is of interest, because it provides a broad snapshot of ECA involvement among young people in society, and because such representative snapshots of current patterns of ECA participation can serve as a baseline for monitoring changes over time. As such, it can practically inform program development and provision on the ground in communities [20–22].

### Conceptual perspectives on ECAs

ECAs are commonly understood as organized activities that take place during out-of-school hours, are voluntary, have regular scheduled meetings, offer adult supervision, include developmentally appropriate rules for participation, and have an overall goal of promoting positive development in one or more domains [1,5]. In a positive youth development (PYD) framework, extracurricular engagement is considered an ecological asset that contributes to thriving by providing a context for pursuing personal interests, engaging with purpose, and developing skills and competencies [23,24], and by enhancing young people’s sense of belonging through the formation of positive relationship with peers and adults [25,26]. In fact, in a recent nationally representative research study with youth, participation in ECAs was linked to having friendships across different social networks, supporting the assumption that ECAs can be a way of promoting young people’s social integration [26].

Participation in ECAs is a multidimensional construct that includes breadth (number of different activities children engage in), intensity (time devoted to the activity), duration (number of months/years children participate), consistency (degree of regular participation), and type of engagement (social, emotional, physical, cognitive) [19,27]. An overall positive relation has been found between greater involvement in ECAs and a range of positive developmental outcomes [5,26,28–32]. For example, among grade 7 to 11 students, intensity in extracurricular involvement, duration, and breadth were positively related to academic adjustment, psychological competencies and positive engaging in positive peer relationships [30].

### Types of ECAs and PYD

Different types of ECAs are thought to have different “nutrients” that contribute to positive development in different ways [6]. Whereas benefits of programs can vary even within a category (e.g., within sports, the benefits of swimming differ from the benefits of playing hockey), several scholars have theorized key affordances of specific groups of ECAs. For instance, educational programs predominately afford educational and academic enrichment that extends learning in school or provides learning opportunities that are not offered through schools (e.g., learning a specific language) [6]. Participation in sports typically affords taking initiative and physical training [33] and—for team sports specifically—managing social relationships with team mates, practicing social skills, and coping with the emotional experiences of winning, losing and the pressures of competing [34]. Participation in the arts affords practicing of precision in focus and detail, creative expression, and the accumulation of focused practice that results in a final product to be showcased [35]. Music programs often provide young people with opportunities for cognitive learning (e.g., understanding and producing the structure and patterns in music), improving music-specific skills, and for expressing and communicating emotions (e.g., music can be used as a means to create, enhance, sustain and change states and moods in a person) [36]. Even though these are not exhaustive descriptions of program affordances and variations prevail within broader activity categories, they can be considered to be important program-specific elements that are theorized to contribute to the link between different ECAs and PYD outcomes.

A large number of rigorous studies have examined the benefits of sports-related ECAs on PYD [37]. For example, results from longitudinal studies comparing participants in organized sports to non-participants have found a wide range of benefits from sports participation, including better academic achievement, improved relationships with peers and adults, and higher self-esteem [28,30,31,38,39]. Additionally, a systematic review of 30 studies that investigated the psychological and health benefits of sports participation in youth (of which 21 studies were conducted cross-sectionally) found that participation in sports was associated with fewer depressive symptoms, improved self-esteem, and better social interactions [40]. The review also revealed that these positive associations were strongest for team sports compared to individual sports; the authors explained this finding with the social learning and belonging aspects of participation in team sports.

### Portfolios of extracurricular engagement among youth

Even though the majority of existing research on ECAs has taken a variable-centered approach, research using person-centered approaches by establishing activities portfolios is growing. Person-centered approaches sort children and youth into clusters based on the types and combinations of activities they are engaging in [17,41–43]. ECA profiles thus consider the breadth, types, and combinations of activities [42].

In a US-based study of 1,711 10- to 18-year-old adolescents, five different ECA profiles were identified: adolescents who were engaged in (1) sports, (2) sports plus other activities (referred to as sports plus), (3) school groups (i.e., school-based clubs excluding sports), (4) religious groups, and (5) students with low or no involvement in activities [17]. The authors found that belonging to the sports only cluster was associated with better developmental outcomes (measures included academic competence, connectedness, and positive behaviors) compared to the cluster of non-participants. Adolescents in the sports plus cluster had higher scores on positive outcome measures than any of the other profile groups. In a US-based study using data from 14,411 6^th^ to 12^th^ grade students in the National Longitudinal Study of Adolescent Health, six activity profiles were found: (1) sports only, (2) academic programs only (e.g., French club), (3) school-specific activities only (e.g., school newspaper), (4) performance-focused programs only (e.g., drama), (5) multiple activities (i.e. a combination of at least 2 activities), and (6) nonparticipation [42]. Females were more likely to participate in school-based and performance activities whereas boys were more likely to participate in sports. Upper middle-class adolescents were more likely to be involved in multiple activities than those from lower socioeconomic backgrounds. Profile membership was not linked to developmental outcomes in this study. A third study based on data from 1,357 youth in the 4-H Study of Positive Youth Development in the US [44], youth across all sports-prominent activity patterns reported higher on measures of PYD (i.e., confidence, competence, character, caring, connection) compared to non-sports youth. However, PYD was highest in clusters in which sports participation was combined with multiple other types of ECAs (e.g., performing arts, religious groups, youth development programs) indicating the link between a “highly engaged” profile in youth and positive developmental outcomes.

Last, six activity profiles were identified in a study with 918 16-to 17-year old adolescents in Maryland [41], including involvement in (1) several activities, (2) sports, (3) school-clubs, (4) volunteering, (5) paid work, and (6) no activities. Controlling for families’ socioeconomic background, being highly involved was associated with better outcomes on measures of academic achievement, resilience, and internalizing/externalizing symptoms. Low levels of ECA participation were related to low levels of positive development. Adolescents involved in sports, along with the highly involved group and those involved in school-clubs scored highest on psychological resilience; highly involved adolescents and those involved in sports scored lowest on internalizing problems.

Overall, these studies suggest participation versus non-participation as well as participating in different combinations of activities is associated with differences in developmental outcomes in young people. It is important to consider that different studies may identify different ECA profiles based on where the research was conducted, which age group was sampled, and what measures were used to assess ECA participation. Different types of activities tend to be available for different age groups as well as across regions/cultures (e.g., engagement in religious programs is more likely in areas with a high degree of religious groups’ activities and engagement) and studies that break down activities in more nuanced categories (e.g., “art program” versus “fine arts” or “performing arts”) can yield a more fine-grained picture. To date, the majority of studies on extracurricular involvement have focused on older adolescents; common portfolios in middle childhood still need to be examined, particularly in the Canadian context.

### The present study

The goal of the present study was to identify profiles of extracurricular engagement in a population of 4^th^ grade students attending public schools in BC, Canada, and to examine whether membership in a profile group was associated with differences in children’s wellbeing (i.e., optimism, satisfaction with life, self-concept) and perceived overall health. Based on previous research using person-centered analyses in this age group, we expected to find a group of children who would be highly engaged in a variety of ECAs, a group who would primarily be engaged in sports, and a group who would be low on extracurricular engagement [17,43]. The identification of additional ECA profile groups was an explorative research question, given that extracurricular activity involvement and types of activities vary according to age group and across regions and cultures. A wide range of studies have found positive associations between participation in ECAs and PYD; thus, we expected that wellbeing and health would be higher among children who participated in activities compared to those who did not. We further explored wellbeing and health differences associated with additional ECA profile groups. Analyses included children’s gender and their reports of first language learned at home (English only versus other) as demographic predictors for ECA profile group belonging, and as control variables when comparing wellbeing and health across the different clusters. This study draws from a large cross-sectional population-level data set of grade 4 students’ self-reports collected in public school districts in BC.

## Material and methods

### Participants

A total of 27,121 4^th^ grade students in 490 schools across 28 public school districts in BC, Canada, were included in the present study. All children answered questions on the Middle Years Development Instrument (MDI), a population-level self-report survey that has been implemented annually with grade four students in participating school districts in BC [45,46]. Children were on average 9.20 years old (*SD* = .54), 51% male and 57.45% reported English as a first language learned at home. Other first languages learned comprising more than 1% included Cantonese, Mandarin, Tagalog, French, Spanish, Hindi, Punjabi, Japanese, Korean, and Aboriginal languages; BC is a culturally diverse province as reflected by the ethnic diversity of the sample in this study.

### Procedures

The MDI was implemented in participating school districts at a population level. All grade 4 students in participating schools were included unless their parents actively withdrew them from the project or they were not present on the day of implementation [45,46]. The present study includes data from six years of implementations (i.e., data were collected once per year from 2010–2016; all grade 4 students in a given year were eligible to participate). For the present study, data from all six years were aggregated to one large cross-sectional data base. Due to administrative reasons, until the 2013–14 school year, the MDI was administered by teachers or other school staff (e.g., principals) in January or February; starting in the 2014–15 school year, MDI administration took place in November/December. A passive consent procedure for student participation was used. Teachers/school staff were provided with materials that prepared them for the survey implementation, including a written manual and videos describing how to administer the MDI. From the manual, they read a verbal assent script that informed students that participation was voluntary, their responses were confidential, and that they could withdraw from the survey at any time. The average student participation rate across school districts was 84%. In order to minimize potential biases associated with variability in children’s reading abilities, teachers read MDI survey questions aloud in the classroom. The MDI was administered electronically or on paper over one to two 40-minute class periods. Analyses examining differential item functioning and data missingness for data collected electronically versus on paper questionnaires found no significant differences. This research was approved by the university human subjects institutional review board of the University of British Columbia, and by the administration of each participating school district.

### Measures

All measures were administered as part of the MDI, a child self-report survey that consists of 8 demographic questions and 77 items assessing children’s social support from adults and peers, psychological wellbeing, physical health, as well as how students spend their time outside of school [45,46]. Evidence for the reliability, factor structure and convergent and divergent validity of MDI sub-scales has been demonstrated in previous research in the Canadian context [45]. The reliability and validity of MDI measures have also been supported in a recent study with approximately 29,000 children between the ages 10 and 15 in the Australian context [47]. The specific measures we draw from in the present study are described below.

#### Demographics

Children reported on their gender (boy or girl), age and what was the first language they learned at home. Information about first language learned at home was used as an indicator for cultural background. Responses were categorized into “English only” and “Other” (this included children reporting both another language than English and English as first languages learned).

#### ECAs

Children reported on the number of days in the past week (ranging from “never” to “5 times per week”) on which they participated in each of the following ECAs during the after school hours (i.e., from about 3:00pm to 6:00pm): (1) educational lessons or activities (e.g. tutoring, math, language school), (2) art or music lessons, (3) youth organizations (e.g., Scouts, Girl Guides, Boys and Girls Clubs), (4) individual sports with a coach or instructor and, (5) team sports with a coach or instructor. Responses to these five items were dichotomized: no participation was coded as 0 and participation on one or more days in the week was coded as 1. Creating binary variables for participation in ECAs serves the purpose of distinguishing between participants and non-participants for each activity; this approach has previously been used in research identifying patterns of ECA participation [18]. The percentage of children who reported participation in an activity at least once in the past week was 35% for educational activities, 40% for arts/music, 19% for youth organizations, 48% for individual sports, and 45% for team sports.

#### Wellbeing

Wellbeing was assessed using the life satisfaction, self-concept and optimism sub-scales of the MDI. Sub-scales were adapted from original scales in the process of developing the MDI [46]. Items for all three measures were rated on a scale from 1 (“disagree a lot”) to 5 (“agree a lot”). Life satisfaction was measured with the 5-item Satisfaction with Life Scale-Adapted for Children [48] (Sample item: “In most ways my life is close to the way I would want it to be”). Internal consistency of the scale was good (alpha = .80). Life satisfaction scores ranged from 1 to 5 in this study, with a mean of 4.10 (*SD* = 0.84). Optimism was measured with an adapted 3-item version of the Optimism subscale in the Resiliency Inventory [49]. Sample item: “I start most days thinking that I will have a good day.” Internal consistency was acceptable for this scale (alpha = .68). Optimism scores ranged from 1 to 5 in this study, with a mean of 4.05 (*SD* = 0.85). Self-concept was measured with an adapted 3-item version of the Self-Description Questionnaire [50]. Sample item: “In general I like the way I am.” Internal consistency for this scale was satisfactory (alpha = .73). Self-concept scores ranged from 1 to 5 in this study, with a mean of 4.40 (*SD* = 0.71).

#### Perceived health

Perceived health was measured with a single item (“in general, how would you describe your health”) adapted from the Youth Health Survey [51]. Children responded to this item on a scale from 1 (“poor”) to 4 (“excellent”). Scores ranged from 1 to 4 in this study, with a mean of 3.42 (*SD* = 0.66). Single item measures of self-reported global health have been shown to exhibit adequate convergent and discriminant validity and have been shown to be sensitive to change in health status over time [52].

### Data analytic approach

Missingness in our dataset ranged from 2% to 4.1% across models. Previous research has shown that a missingness rate of 5% or below is inconsequential (Schafer, 1999). After listwise exclusion of missing data, final sample sizes were *n* = 26,020 for predicting life satisfaction, *n* = 26,306 for predicting optimism, *n* = 26,450 for predicting self-concept, and *n* = 26,575 for predicting perceived overall health. We employed Latent Class Analysis (LCA) to explore children’s profiles of engagement in ECAs. LCA is a statistical tool for identifying underlying subtypes; latent classes are measured indirectly through response patterns across different observed variables [53]. LCA seeks divisions that maximize the differences between clusters and minimize differences within clusters. Analyses were conducted with Mplus14 [54] with full information maximum likelihood (FIML) as estimation method, by including four ECA items in the LCA: participation in (1) educational lessons or activities (2) art or music lessons (3) individual sports with a coach or instructor (4) team sports with a coach or instructor. The item measuring children’s participation in youth organizations (e.g., Scouts, Girl Guides, Boys and Girls Clubs) was not included in the final LCA because more than 80% of children endorsed that they did not participate in such activities. Such homogeneity led to non-salient classification pattern across identified latent classes and the item did not contribute to a classification solution in our model.

We selected the best fitting model resulting from LCA (i.e., the number of latent classes representing ECA profiles that could be identified) through a 2-step process. First, we determined the model with the smallest Akaike Information Criterion (AIC), Bayes Information Criterion (BIC), and adjusted-BIC values. Second, using the Lo-Mendell-Rubin likelihood ratio test (LMT-LRT) and the bootstrap likelihood ratio test (BLRT), we determined whether additional latent classes offered a significant improvement over solutions with fewer latent classes [53,55]. We present a) model fit evaluation information that guided the selection of the final model b) estimated parameters of class prevalence (i.e. the probability of class membership) and c) item response probability (i.e. probability of item response conditional on class membership) [56]. After establishing the latent profiles, we examined the representation of English only versus other language backgrounds, and gender across the latent profiles. We tested whether the proportions of English only versus other, and female versus male differed significantly across the latent profiles; we then conducted multinomial logistic regression analyses in which first language status or gender predicted the odds of class membership to determine whether the differences in proportions were statistically significant. We report the estimated regression coefficients, standard errors, odds ratios and 95% confidence intervals from these analyses. Finally, we examined the association between latent class membership and children’s reported wellbeing (optimism, self-concept, life satisfaction and perceived health) by conducting four ANCOVA analyses with gender and English only/other entered as covariates. We conducted analyses involving ANCOVA in SAS 9.4 PROC GLM and report the F-statistics, p-values, effect sizes, and the adjusted means and 95% confidence intervals for these means for each wellbeing indicator across the latent classes.

## Results

### Identification of latent classes

For the latent class analysis, a 4-class solution was the best fit based on the data in the present study (see Table 1). It had better fit indices and presented a significant improvement compared to the 3-class solution. The 5-class solution had a slightly poorer fit and the LMT-LRT and the BLRT did not indicate a significant improvement over the 4-class solution.

**Table 1:** Model fit indices.

Model	n-par^a^	AIC	BIC	adj-BIC	LMT-LRT	BLRT
2-Class	9	138607.23	138681.11	138652.50	< .00001	< .00001
3-Class	14	138388.51	138503.43	138458.94	< .00001	< .00001
4-Class	19	138261.48	138417.43	138357.05	< .00001	< .00001
5-Class	24	138271.48	138468.47	138392.20	0.4191	>.9999

^a^ n-par = Number of parameters in the model

**Fig 1:**
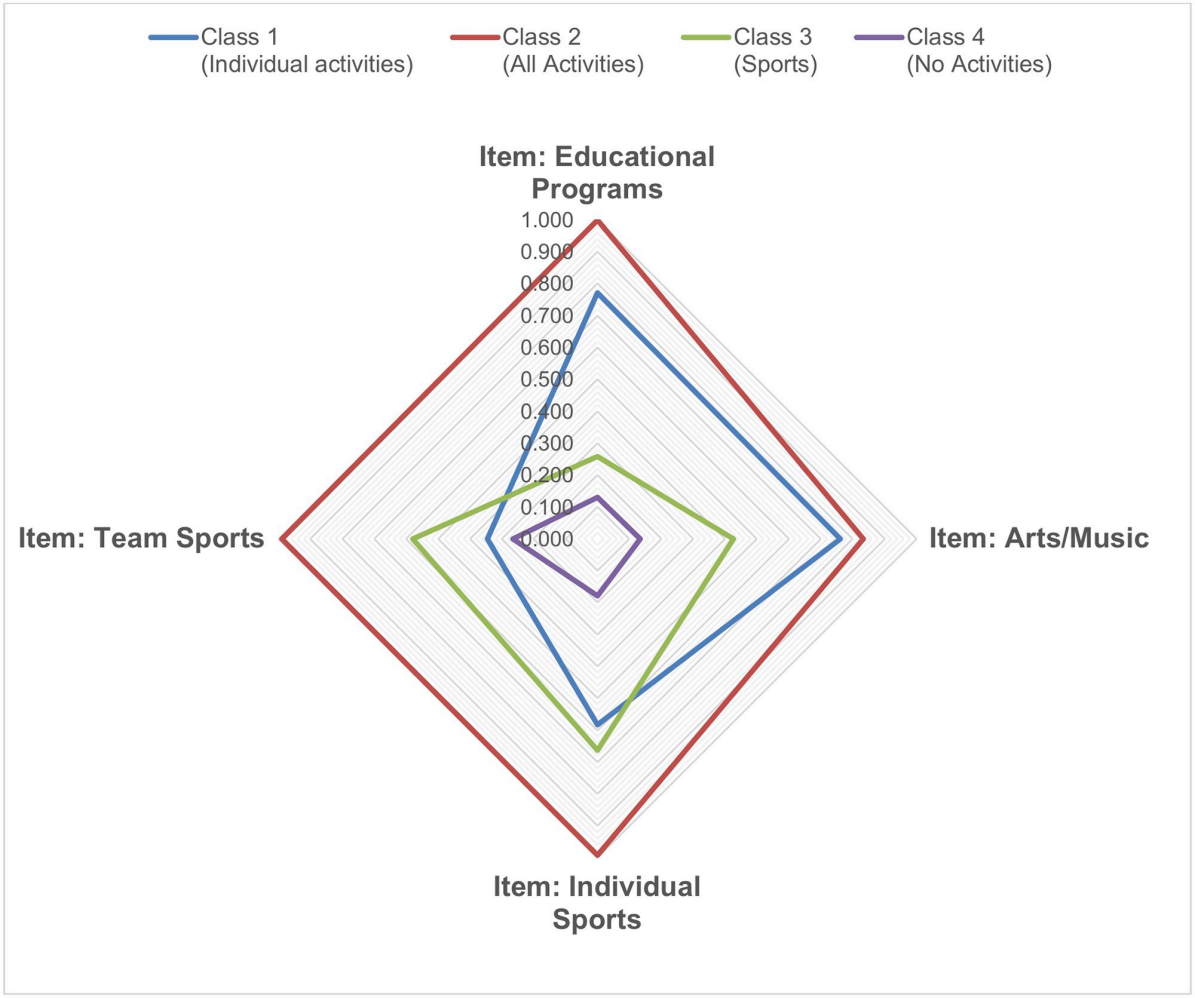
Extracurricular activities profiles.

Table 2 shows the item response probabilities and latent class prevalences of the 4-class model for each latent class. Fig 1 visualizes these results. Children in Latent Class 1 (“Individual activities”) were more likely to participate in individual programs and less likely to participate in team sports. Children in Latent Class 2 were more likely to participate in all four ECAs (“All Activities”). Children in Latent Class 3 (“Sports”) were more likely to participate in individual sports and team sports. Children in Latent Class 4 (“No Activities”) were more likely to participate in none of the ECAs. In terms of class prevalence, the two predominant subgroups were children who participated in individual and in team sports (40.42%; *n* = 10,961) and children who did not participate in any ECAs (39.15%; *n* = 10,619). Children who participated in all types of activities (9.2%; *n* = 2,495) and children who participated mostly in educational programs, music/arts programs, and individual sports (11.23%; *n* = 3,046) made up a relatively small proportion of the sample.

**Table 2:** Latent class prevalence and item response probabilities for extracurricular activity items.

	Item: Educ. Programs	Item: Arts/Music	Item: Ind. Sports	Item: Team Sports	n	proportion
**Class 1** (Individual activities)	0.771	0.761	0.583	0.345	3046	11.23%
**Class 2** (All)	0.999	0.833	0.992	0.989	2495	9.20%
**Class 3** (Sports)	0.258	0.426	0.663	0.579	10961	40.42%
**Class 4** (None)	0.130	0.134	0.179	0.265	10619	39.15%

### Representation of demographics in extracurricular classes

We compared the probabilities of ECA latent class membership by first language learned (English only versus other) and gender (female versus male). In the clusters “Individual activities” and “All Activities”, children reporting a language other than English as their first language learned at home were over-represented [P_other_(Individual activities) = .246; P_other_(All Activities) = .082] compared to children who reported only English as their first language [P_English_ (Individual activities) = .109; P_English_(All Activities) = .062]. The pattern was reversed for the clusters “Sports” and “No Activities”, with a language other than English being under-represented [P_other_ (Sports) = .315; P_other_ (No Activities) = .357] compared to English only as first language [P_English_ (Sports) = .411; P_English_ (No Activities) = .417]. Table 3 shows the results of the multinomial logistic regression analysis in which first language learned predicted odds of class membership, compared (with “No Activities” as a reference group). Children reporting English only as their first language learned at home exhibited significantly lower odds of membership in the “Individual activities” and the “All Activities” clusters, compared to children reporting a language other than English. By contrast, first language learned did not significantly predict odds of membership in the “Sports” cluster.

**Table 3:** Results of multinomial logistic regression analysis with demographics predicting latent class membership.

	Est.	s.e.	Odds Ratio	95% LL	95% UL
**First language learned**					
Individual activities Eng. /other	-0.967	0.068	0.380	0.332	0.434
All Eng. /other	-0.422	0.073	0.643	0.557	0.741
Sports Eng. /other	0.110	0.063	1.116	0.986	1.263
**Gender**					
Individual activities Male /Female	-0.844	0.066	0.430	0.378	0.489
All Male /Female	0.423	0.086	1.527	1.290	1.807
Sports Male/Female	-0.680	0.062	0.507	0.449	0.572

*Note*: The latent class “None” served as a baseline in the analyses; Other = 0, English only = 1; Female = 0, Male = 1

Regarding the probabilities of ECAs latent class membership across genders (female versus male), girls were over-represented in the clusters “Individual activities” and “Individual & Team Sports” [P_female_ (Individual activities) = .208; P_female_ (Sports) = .429] compared to boys [P_male_ (Individual activities) = .129; P_male_ (Sports) = .314]. Girls were under-represented in “All Activities” and “No Activities” [P_female_ (All Activities) = .044; P_female_ (No Activities) = .319] compared to boys [P_male_ (All Activities) = .096; P_male_ (No Activities) = .416]. Table 3 shows the results of the multinomial logistic regression analysis in which gender predicted odds of class membership (with “No Activities” as reference group). The results showed that males had significantly lower odds of membership in “Individual activities” and “Sports”, and significantly greater odds of membership in “All Activities”. Interactions between gender and home language predicting class membership were examined and were not significant.

### Associations between class membership and wellbeing

Table 4 shows the results of the ANCOVA analyses and the adjusted means and 95% Cis for each wellbeing indicator across the four latent classes. Results indicated that children’s mean levels of optimism, satisfaction with life, self-concept and perceived health differed statistically significantly across latent extracurricular classes. Post-hoc analyses with Tukey HSD adjustment (see 95% Cis in S1 Table) indicated that children in the clusters “Individual & Team Sports” and “All activities” exhibited significantly higher mean scores across all four wellbeing indicators than children in the clusters “Individual activities” and “No Activities”. Effect sizes (see S1 Table) ranged from .1 to .2, which can be considered small effects (Cohen, 1988). There were no significant differences between children in “Individual activities” and “No Activities” for any of the wellbeing indicators; children in “All Activities” did not differ significantly from children in “Sports” regarding their mean scores in satisfaction with life, self-concept, and health. Children in “All Activities” had significantly higher mean optimism scores than children in “Sports” but the size of this effect was very small (d = .04).

**Table 4:** Results of ANCOVA with latent class membership predicting well-being, adjusted for gender and home language.

		Optimism			Life Satisfaction			Self-Concept			Perceived Health	
	*M*	*LL*	*UL*	*M*	*LL*	*UL*	*M*	*LL*	*UL*	*M*	*LL*	*UL*
1. Individual activities	4.00	3.97	4.03	4.04	4.01	4.07	4.34	4.31	4.37	3.38	3.36	3.40
2. All	4.15	4.11	4.18	4.15	4.11	4.18	4.24	4.40	4.45	3.48	3.45	3.50
3. Sports	4.09	4.07	4.10	4.14	4.13	4.16	4.44	4.43	4.46	3.46	3.44	3.47
4. None	3.98	3.97	4.00	4.05	4.03	4.06	4.35	4.34	4.36	3.26	3.35	3.38

*Notes*. M = mean, LL = lower limits of 95% Confidence Interval, UL = upper limits of 95% Confidence Interval.

Optimism: *F*(3, 26300) = 44.34, *p* < .0001; Life Satisfaction: *F*(3, 26014) = 32.53, *p* < .0001;

Self-concept: *F*(3, 26444) = 47.44, *p* < .0001; Perceived health: *F*(3, 26569) = 46.78, *p* < .0001;

## Discussion

The goal of this study was to identify profiles of extracurricular engagement in a population of grade 4 children in BC, Canada, and to investigate whether membership in a specific profile group was linked to differences in children’s positive wellbeing and health, taking into account gender and home language background. Based on self-reports from more than 27,000 children, we identified four distinct profiles that reflected (1) no involvement, (2) involvement in all activities, (3) involvement in individual programs (i.e., educational programs, arts/music, and individual sports), and involvement in individual and team sports during extracurricular hours. Most children fell into the clusters of non-participants (39%) and sports (40%). The smallest cluster consisted of the children who participated in all activities (9%), followed by those who participated in activities within the education lessons, arts/music, and individual sports domains (11%). These profiles were overall in alignment with previous research that has identified clusters of youth that were highly involved, uninvolved, and predominately involved in sports [17,43,44], suggesting that ECA grouping may begin as early as in middle childhood and represents the experiences of many children and adolescents in North America. The finding that a relatively large number of children (i.e. 39%) were uninvolved in ECAs is also consistent with previous research. Specifically, the group of uninvolved youth made up 37% [17] and 34% [57] of the samples in previous studies. Replicating this finding at a population level emphasizes the need for understanding the reasons and potential barriers (e.g., costs, access, transportation, availability, lack of interest, preference for unstructured activities) that prevent a large number of children from participating in ECAs.

A major finding in the present study was that across the profile groups, wellbeing (i.e. optimism, satisfaction with life, self-concept) and health were highest in children who were involved in all ECAs and those who were involved in sports, compared to children who were uninvolved and those who predominately participated in educational programs, arts and music, and individual sports. There were no significant differences regarding the wellbeing and health outcomes when comparing children who participated in all activities to those who predominately participated in sports (except for a very small effect for higher optimism among children in the “all activities” cluster compared to those in the “sports” cluster). This finding is interesting because it suggests that participation in sports in particular was a key ingredient in the identified clusters in the present study that was associated with better wellbeing and health.

Previous research has underscored the benefits of sports for wellbeing and positive development. For example, a systematic review of extracurricular participation in sports during childhood and adolescence found that sports engagement potentially improves internalizing problems and enhances positive mental wellbeing [58]. The benefits of team-based sports have been highlighted in particular in a study that examined the psychological and social health benefits of sports in childhood and adolescence [40]. This finding is also in line with our research, since in the present study, higher levels of wellbeing were not associated with participation in the cluster of individual activities that included individual sports.

However, our finding highlighting higher levels of health and wellbeing for children in the sports group also contrasts two previous person-centered studies on extracurricular engagement [17,44]. Specifically, Linver and colleagues found that youth who participated in sports in addition to other activities (described as “sports plus”) fared overall better regarding developmental outcomes than those who only participated in sports (17). Similarly, Zarrett and colleagues found that PYD outcomes were higher among youth who participated in multiple activities, including sports than among the sports only group (46). Several explanations for these differences in findings can be considered. First, in the present study, we focused on measures of emotional wellbeing and health as positive markers of development. In comparison, Linver and colleagues (17) assessed youths’ academic competence, confidence, connectedness, and positive and negative behaviors; Zarrett and colleagues [44] assessed youths’ PYD competencies (confidence, competence, character, caring, and connection), youth’ contributions (e.g., helping and serving in the community), risk behaviors, and depression as developmental outcomes. As such, it is possible that the link between sports and wellbeing and health is stronger than the link between sports and academics and PYD-competencies. Second, the present study included only grade 4 students (approximately age 9), representing the developmental stage of middle childhood whereas Linver and colleagues drew from a combined sample including youth with an age-range of 10 to 18 and Zarrett and colleagues assessed PYD in a sample of grade 7 youth. It is possible that the links between ECAs and positive outcomes vary across ages and developmental stages. Last, the present study focused on organized extracurricular activities only, whereas Zarrett and colleagues [44] study took a broader approach and also included other dimensions of out-of-school time, including paid work, involvement in school clubs, and religious activities. For the present study, these dimensions were not relevant (i.e., paid work does not apply yet and school clubs are more commonly found in older age groups in BC public schools).

A second important finding in the present study was that children who were a part of the “no activities” cluster did not differ significantly regarding wellbeing and health outcomes from those who predominately participated in educational programs, arts/music, and individual sports. This finding can be interpreted in several ways. First, it is likely that the activities in this cluster (e.g., taking part in a tutoring program, learning to play an instrument, carrying out an individual sport) have a strong individual focus, rather than a social focus. Previous research has highlighted that some of benefits of ECA participation are specifically tied to the social interactions that children are gaining with peers through the activities (26, 27). Further, educational lessons in this study were described as “tutoring, math, and language school”; arts and music lessons included the examples of “drawing, painting, and playing a musical instrument”. Whereas a systematic review has suggested positive effects of extracurricular academic tutoring for middle school students’ academic achievement [59,60], research that investigates its relation to wellbeing and perceived health is still lacking. In a study with grade 8 students, Luthar and colleagues found that high levels of participation in extracurricular academic programs in combination with parental pressures around achievement were linked to lower levels of positive adjustment in 8^th^ grade girls [61]. One interpretation of our finding could thus be that children who predominately pursue the combination of activities involving educational programs, music/arts, and individual sports may engage in some of these activities to meet parental expectations, and that this was linked to lower levels of wellbeing and health. Since we did not measure whether children self-selected the activities or whether participation was decided by parents, future research is needed to confirm this potential link.

The findings in the present study had small effect sizes. Nevertheless, their importance should not be disregarded. The present study was based on a very large population-level sample. Methodological research has shown that population-level and large-scale samples (i.e. *N* > 2,000) tend to yield smaller but more accurate effects sizes in research than studies using small sample sizes [62]. The use of so-called bench-mark sample sizes—effect sizes commonly found in one’s area of research—has also been recommended. The effect sizes found in the present study correspond to the small effect sizes in previous extracurricular involvement research [17,28]. In an editorial on ECA research, Simpkins [63] argued that even small effect sizes indicating the positive links between ECAs and positive outcomes are important because effects are cumulative in nature of development where small changes compound over contexts and time.

In sum, the present study highlighted extracurricular sports activities as a key ingredient that was positively associated with ECAs and wellbeing and health. Being engaged in other activities in addition to sports was not linked to higher wellbeing and health self-reports. Participating only in educational programs, arts/music, and individual sports was associated with comparable levels of wellbeing and health to not participating in ECAs. Non-participants and sports participants were the largest clusters in the present study, a finding that is consistent with other research in North American contexts. Revealing these patterns in a population-level sample of grade 4 students is important because it suggests that participation patterns are established as early as in middle childhood. A large majority of research in this domain has been conducted with older adolescents and high school students in particular. Emphasizing a link between ECAs and positive wellbeing and health is critical because it contributes to identifying ways for promoting the positive development of the whole child with a specific focus on extracurricular programs that take place in the community [64]. Many of the previous studies have been conducted in the context of the United States, whereas the present study adds to the smaller number of studies conducted in the Canadian context.

### Limitations and future directions

Several limitations need to be noted. Given that the present study drew from a large data set collected at the population level for the purpose of monitoring child development in schools and communities across a broad range of domains, some detailed information about ECAs (e.g., quality of the programs, the specific types of group/individual sports or music programs, the duration per meeting, and since when and how regularly children had been participating) was not available. Future research needs to include more finely nuanced questions about program type and characteristics. Similarly, additional demographic data (e.g., socioeconomic backgrounds of families, parent education) and information about parental expectations and whether children self-selected activities were not available and could therefore not be taken into account. Additional information is necessary to distinguish the different sub-groups of children who are non-participants (i.e., those who do not wish to participate from those who cannot participate because they face different types of barriers). The present research was focused on children in grade 4 in public schools in BC. Future research needs to explore profiles that may emerge in other age groups, whether profiles tend to be stable over time.

## Supporting information

S1 Table Table with results of Post-hoc analyses of pairwise comparisons. (PDF)
