# Supplementary material for: Extracurricular activity profiles and wellbeing in middle childhood: A population-level study
Source: PLoS One. 2019 Jul 10;14(7):e0218488. doi: 10.1371/journal.pone.0218488 (PMC6619656; doi:10.1371/journal.pone.0218488)
Supplement: S1 Table — (PDF) [file pone.0218488.s001.pdf]

S1 Table. Results of Post-hoc analyses of pairwise comparisons

		Optimism				Life Satisfaction				Self-Concept				Perceived Health			
CL(i) - CL(j)		*M(i)-M(j)*				*M(i)-M(j)*				*M(j)-M(j)*				*M(i)-M(j)*			
i	j	*MD*	*LL*	*UL*	*d*	*MD*	*LL*	*UL*	*d*	*MD*	*LL*	*UL*	*d*	*MD*	*LL*	*UL*	*d*
Ind. activities	All	-0.14	-0.20	-0.08	-.15	-0.11	-0.17	-0.05	-.12	-0.09	-0.14	-0.04	-.10	-0.10	-0.14	-0.05	-.14
Ind. activities	Sports	-0.09	-0.13	-0.04	-.11	-0.10	-0.15	-0.06	-.14	-0.10	-0.14	-0.07	-.18	-0.08	-0.11	-0.04	-.13
Ind. activities	None	0.02	-0.02	0.07	.03	-0.01	-0.05	0.04	-.02	-0.01	-0.05	0.03	-.02	0.02	-0.02	0.05	.01
All	Sports	0.06	0.01	0.11	.04	0.00	-0.04	0.05	-.02	-0.02	-0.06	0.02	-.07	0.02	-0.02	0.06	.03
All	None	0.16	0.11	0.21	.17	0.10	0.05	0.15	.10	0.08	0.04	0.12	.10	0.11	0.07	0.15	.16
Sports	None	0.11	0.08	0.14	.14	0.10	0.07	0.13	.12	0.09	0.07	0.12	.15	0.09	0.07	0.15	.15

Note. Ind. Activities = Individual activities (i.e., Educational programs, Music/Arts, Individual Sports).
Tukey HSD adjustment was used for multiple pairwise comparisons.
Underlined values represent statistical group difference.
CL = (Latent) Classes, M = mean, MD = mean difference, LL = lower limits of 95% confidence interval, UL = upper limits of 95% confidence interval;
d = Cohen's d
